# Mental health care policy environment in Rivers State: experiences of mental health nurses providing mental health care services in neuro-psychiatric hospital, Port Harcourt, Nigeria

**DOI:** 10.1186/1752-4458-7-8

**Published:** 2013-02-15

**Authors:** Izibeloko Omi Jack-Ide, Leana R Uys, Lyn E Middleton

**Affiliations:** 1 Faculty of Nursing, Department of Mental Health, Niger Delta University, Wilberforce Island, Bayelsa, Nigeria; 2 School of Nursing and Public Health, University of KwaZulu-Natal, Durban, South Africa

**Keywords:** Mental health policy, Mental illness, Primary mental health care, Professional experiences

## Abstract

**Background:**

Mental health services for Rivers State and surrounding States in the Niger Delta region of Nigeria are provided only at the neuropsychiatric Rumuigbo Hospital in Port Harcourt City, Rivers State, Nigeria. The study explored mental health nurses’ experiences of providing mental health services at the hospital in an attempt to understand policy implications, identify difficulties and challenges of delivering mental health care services.

**Methods:**

A qualitative study using in-depth interview was conducted among 20 mental health nurses working at the neuropsychiatric Rumuigbo Hospital. This was reviewed within the Townsend mental health policy template of context and resources domains.

**Results:**

A lack of political support and senior position in the Ministry of Health hinders service delivery, the prevalence of institutionalized stigma, a lack of training, and system failure to provide services at all levels of care is hampering service delivery. The inadequate allocation of resources for hospital renovations and equipment is preventing appropriate client care, as does the lack of funding for drugs, the cost of which makes them unaffordable, affecting clients staying on treatment.

**Conclusion:**

Education and training of mental health care professionals should be given priority to remedy human resource shortage, provide incentives to motivate health professionals for psychiatric practice, and move toward decentralization of care into general health care services. Information should be provided at all levels to overcome the myths surrounding the causes of mental illnesses, to reduce stigma and discrimination of the affected and their families.

## Background

Mental disorders account for a significant and growing proportion of the global burden of disease, yet remain a low priority in many low- and middle-income countries [1]. It is anticipated that by 2030, mental health problems will constitute 15% of the global burden of disease [2]. In spite of the growing burden of mental disorders and the resultant level of suffering for individuals and society, efforts to address it remain unsatisfactory [3-5]. The international trend is no longer to provide mental health only at institutional levels of care, but to decentralized these to community-based mental health care services [6,7]. The World Health Organization (WHO) proposes the integration of mental health services into primary health care, these being supported by other levels of care [8]. It is anticipated that this model has the potential to reduce stigma, address health worker shortages, and improve access to services by removing barriers to early treatment and support [7]. Failure to addresses the problems associated with caring for people with mental health problems can increase poverty in families, and contribute to the poor attitude of policy makers to issues of mental health [9,10].

A nation’s health policy affects the mental health of its citizenry, and countries need to establish mental health systems that are accessible, equitable, efficient and financially supportive of their needs and expectations [11-13]. In 1981, Nigeria implemented Primary Health Care (PHC) as its national health policy, and adopted mental health as its ninth component in an attempt to provide care and improve the quality of life of persons with mental illness [14,15]. In 1991 a National Mental Health Policy was formulated to ensure comprehensive delivery of mental health services through PHC. While the policy made provision to establish mental health services in rural communities, several years after its adoption this has not happened and the service is still institutionalized. A number of studies done in Nigeria have indicated that this is due to a shortage of appropriately trained professionals, poor knowledge of mental disorders by primary health care workers, and the low priority status of mental health services on policy agendas [16-18].

In Rivers State, Nigeria, treatment for mental illness was free until 1996, when the government realized that patients from neighboring states were benefiting from the program, after which patients were required to pay out-of-pocket with a level of subsidy. The impact of this policy change on the River State inhabitants has not been established nor the implications for the lack of mental health services at PHC level. Hospital records shows an increased of patients from 4000 in 2008 to 9000 in 2009. A study of prevalence of schizophrenia in Rumuigbo Hospital by Afolanyo and colleagues [19] revealed that 7723 patients were admitted in 2005–2009 and the prevalence of schizophrenia was 58.19% of the total admission, the condition being more common with males, and with a higher percentage among those with secondary and tertiary levels of education.

Rivers State is an oil producing region with a population of approximately three million people, with a large proportion being concentrated in a few towns and the state capital. Mental health services are delivered through a single mental health hospital, the neuropsychiatric Rumuigbo Hospital, which provides treatment for all types of mental disorders – neurosis or psychosis, acute or chronic, outpatient and inpatient care. The majority of users access the facility directly due to advice they received from friends and relatives without referral consultations, with only a few being referred from secondary or tertiary facilities. The hospital has no working relationship with other health facilities that provide health care service in Rivers States 23 Local Government Areas (LGAs), none of which provide mental health services. This has resulted in an increased workload for the few mental health professionals at the hospital, nurses forming the bulk of the workforce (Table 1).

**Table 1:** Mental health professionals working at neuropsychiatric hospital

**S/N**	**Categories of professionals**	**Number**
1	Mental Health Nurses	76
2	Psychiatrists	1
3	Other medical Doctors (not specialized in psychiatry)	2
4	Pharmacists	1
5	Psychologists	1
6	Social workers	1

This hospital is located in the State capital, making access to mental health services difficult for people living in rural areas, particularly those living far away. Due to the provision of this service in only one facility in the state, it is important to know how the current mental health policy of centralized care impacts on mental health care professionals, patients and families living with mental disorders from the perspectives of the mental health nurses. The study aims to explore mental health nurses’ experiences of providing mental health services, in an attempt to understand policy implications, identify difficulties and challenges of providing mental health services so as to suggest policy reforms.

## Methods

Purposive sampling was used, and participants were sought across various cadres of mental health nursing staff working at the Rumuigbo Hospital. 20 nurses were recruited to participant in the study via the Department of Nursing Services. The inclusion criteria were that the mental health nurses had to be employed full-time with at least one year experience working at the facility, to ensure that they had adequate experience to participate and understand the mental health policy environment for service delivery. Informed consent to participate in an audio-tape interview was obtained and all participants were advised that they could withdraw from the study at any time.

### Procedure

The study used in-depth semi-structured interviews schedule developed from Townsend et al’s mental health policy template [20] to obtain information about mental health nurses’ experiences of providing mental health care service within the current mental health care policy environment. The model provides a framework for systematic policy evaluation and consists of four domains: context, resources, provision, and outcomes, each of which considers a number of elements that can be used in mental health service assessment and programs implementation. The mental health policy template’s application helps to identify those elements that need to be considered during policy evaluation. In this study, the experiences of mental health nurses of providing mental health care services were explored within the first two domains of context and resources only. The reason being that information derived from these two domains will serve to define the provision and outcomes domains. The context and resources domains highlight factors that drive mental health services activities and programs, and address the influence of cultural beliefs systems on service provisions and funding. The information collected in the context and resources domain serves to inform the action undertaken in the provision and outcomes domains with respect to successes, failures or challenge that impact on service delivery, so as to raise mental health service onto the policy agenda.

The Context Domain and its elements describes how mental health nurses experienced the prevailing social, economic, cultural and political factors that impact on mental health service delivery. The Context Domain consists of four elements; Societal organization and culture, Public policy, Governance and Population need and demand. The Resources Domain describes how mental health nurses experience the resources available to address the needs of service users, inputs that are injected into mental health services to provide mental health care services such as beds, facilities, staff (human resources), medications, transport. The Resources Domain consists of five elements; Financing, Human resource, Physical capital, Consumables and Social capital.

On completion of a consent form, an audio taped interview was conducted in a quiet room within the hospital premises that lasted up to one hour, using open-ended questions that cover the prevailing situation. Participants were encouraged to discussed and reflect upon their experiences and perceptions of the mental health policy environment in which the service is rendered, constraints and shortfalls in the current mental health policy of centralized care and challenges of service delivery. This allowed emerging issues to be clarified with follow-up questions. The sample was a theoretical sample, allowing data collection to continue until data saturation was achieved. For example, for the societal organization and culture in the context domain, the open-ended question was posed, “How is mental health service perceived by the public and other health workers?” Depending on the reply, a follow-up question was posed, such as, “Do they feel any form of discrimination?” The interview schedule was piloted with four nurses and modified for the study (Table 2).

**Table 2:** **Mental health policy template, adapted from Townsend et al.**[20]

**Domain**	**Descriptions and elements**	
**Context**	This describes public policies and governance that define government’s actions and roles towards implementing mental health services/programs, as well as addressing specific factors necessary for mental illness prevention. It comprises four elements.	
	**Elements**	Societal organization and culture
		Public policy
		Governance
		Population need and demand
**Resources**	Resources are those elements that are injected into mental health services to provide care and how these resources impact on service delivery. It comprises five elements.	
	**Elements**	Financing
		Human resources
		Physical capital
		Consumables
		Social capital

### Analysis

The transcripts were transcribed verbatim, analyzed and organized according to the elements of the mental health policy template [20], and were coded according to those themes using the template editing style described by Crabtree and Miller [21]. This process reduced the amount of data being considered at any one time and brought together related pieces of text. QRS NVivo 8 software [22] for analyzing qualitative data was used to facilitate this process. An initial scan of the data highlighted words or phrases used by the participants, and matched them against the domains and elements of the mental health policy template to ensure internal consistency. A coding frame was developed that reflected these issues and was expanded or refined as new issues arose, with related issues being grouped into broader themes. This was followed by finding connection between themes, while emerging meanings were distilled and thematically refined in consultation with co-researchers. The data was then reread and assigned excerpts that illustrated each element of the policy template. Three methods of triangulation were adopted [23]. First, the transcripts were read and discussed by the co-authors developing the analysis throughout the study. Second, theoretical sampling was used until saturation occurred. Third, the emerging analysis was tested in two focus group discussions with 14 of the 20 nurses who had participated in the study. These involved discussions across the various cadre of nurses about their experiences and perceptions of the current mental health policy environment of service delivery. Results from the current study were fed back in these groups interviews.

### Ethics approval

Ethical approval for the study was obtained from the Social Sciences and Humanities Research Ethics Committee of University of KwaZulu-Natal, Durban, South Africa and also from the Ministry of Health and the Ethics Committee of Neuro-Psychiatric Rumuigbo Hospital, Port Harcourt, Rivers State, Nigeria. The study was carried out from February- June 2010.

## Results

The results are presented according to the Context and Resources Domains and their respective elements. The sample was divided equally between men and women, 95% were senior nurses, and 5% were junior staff. Almost half were above 46 years old, and 70% had worked in mental health services for 11 to over 31 years in the facility with average length of service in Rumuigbo Hospital being 20 years (Table 3).

**Table 3:** Demographic details reported by the study participants

**Items**	**Number**	**%**	**Items**	**Number**	**%**
**Occupation**			**Gender**		
Nurse Administrators	10	50	10	Male	50
Chief Nursing officers (CNOs)	7	35	10	Female	50
Assistant CNOs	1	5	**Age**		
Principal Nursing officers	1	5	18–25	1	5
Staff Nurses	1	5	26–35	2	10
**Years of Experience**			36–45	8	40
1–5	2	10	46–55	9	45
6–10	2	10	**Education Level**		
11–20	4	20	Diploma	3	25
21–30	8	40	Tertiary	2	75
31 and above	4	20			

### Context domain

This consisted of four elements that relate to the context in which the health services were provided. The four elements are: Societal organization and culture, Public policy, Governance, Population need and demand.

1) Societal organization and culture

Reports of stigma and negative attitudes towards mental health services among health professionals was reflected in a lack of interest in following a career in mental health practice and may be responsible for the reduced mental health human resource in the State. A Nurse Administrator observed: “*Stigma is one of the major challenges in the profession; most people don’t want to practice in this area because psychiatry is not regarded as important…” (Interview 7).*

All the participants believed that stigma and discrimination of mental disorders contributes to the low status of mental health services in the health policy agenda of the State, thus reflecting the poor state of the facility. A Nurse Administrator, with 30 years working experience at the facility observed: “*The government can tackle and solve our problems if they want to but the issue of negative perceptions about this hospital is a challenge. I think that is the problem, stigma or whatever…even within the health profession stigma is affecting us” (Interview 12).*

2) Public policy

The majority of the participants reported that while the State government was currently renovating all the public hospitals, the psychiatric hospital was not being renovated, reflecting the lack of importance attributed to these services. A Chief Nursing Officer (CNO) observed: “*Psychiatry is always kept at the background, nobody wants to mention psychiatry, nobody wants to associate with psychiatry so that is the problem, until those at the helm of affairs begin to recognize the need for mental health care service, people with mental illness will continue to suffer with no form of service in the communities” (Interview 9).*

The participants held the view that there was a policy failure regarding mental health service provision in both urban and rural areas. The primary mental health policy, if implemented in PHC level, would have resolve most of the difficulties of service users, especially those coming from rural areas. A CNO illustrates: *If mental health service is made available in primary health centers, it will help the people. This is the only hospital serving the State 23 LGA’s and they are all far from Port-Harcourt, apart for a few persons that live within this city environs…it’s very far for majority of service users. (Interview14).*

### Governance

Participants reported that the Ministry of Health (MOH) is responsible for all health care systems, for implementing the primary mental health policy, and carrying out regulatory and supervisory roles in mental health activities and programs. The majority of participants observed the absence of mental health care professionals in PHC clinics, or in schools for mental health assessment and promotion. No posts have been created in the MOH for mental health, and these services are often supervised by officials with other primary duties. Furthermore, the lack of a Director position in the MOH responsible for mental health activities and programs was considered an obstacle to service delivery. A Nurse Administrator elaborates: “*I think its management problem, who is there as the head in MOH for mental health programs? Nobody, there are lots of politics over there, this hospital was built and commissioned in 1977 and until now mental health care service has not taken a proper position, just this hospital for the whole state” (Interview 10).*

### Population need and demand

All the participants identified the need for information about identifying mental illness and how to locate mental health services to be made available to the public. They believe that this would reduce stigma and discrimination of mental disorders, while health education could assist clients with their health needs and provide a support system. Mental health education is currently only provided once the clients are accessing care at the hospital. A CNO elaborates: “*All we do is provide support for clients…we health educate them on how to care for themselves, take their prescribed medication, keep clinic appointments and things to avoid to stay healthy…We feel that is most important and…of course the health talk help them to adjust” (Interview 8).*

The majority of participants observed the need for decentralization of mental health services in primary and secondary facilities that can treat and accommodate all types of mental disorders: neurosis or psychosis, acute or chronic, due to increase in mental illnesses resulting in a greater demand for this single service. They observed that the government is responsible for implementing the primary mental health policy at the PHC level and for increasing access to services. A CNO with over 33 years working experience illustrates: *“Mental illness is on the increase due to economic hardship, every day we have over ten new cases. The kind of patients we have now is different, we were not having acutely ill cases before but now we have more acute cases everyday, the use of the facility has increased” (Interview 20).*

### Resources domain

This domain consist of four elements that deals with resources that are made available to address the needs of service users, these being financing, human resource, physical capital, consumables and social capital.

### Financing

Participants observed that the current health policy environment required all State hospitals to generate funds for their running cost including the psychiatric hospital. The facility is able to procure the medications through revolving loans given by the government to prevent them from being out-of-stock, while patients pay out-of-pocket for the service. A Nurse Administrator elaborated: “*The hospital is not having any external support, the facility is maintained from fees patients pay, all state hospitals for now are autonomous. Autonomous in the sense that the hospital is self sustained by what patients pay for its daily running cost, while government is responsible for staff salaries” (Interview 5).*

### Human resources

Majority of the participants observed that people were recruited and trained in various aspects of mental health practice and posted to work in psychiatric hospital after their training. Government no longer sponsored or trained psychiatric nurses and other mental health professionals which resulted in a decline in the mental health human resource. A Nurse Administrator observed: “*For the past 15 years the Health Management Board or Ministry of Health has not trained psychiatric nurses or any other personals, the last batch sent for training was over 20 years ago. Currently we have one psychiatrist, two physicians and a few others, so when we all retire in a few years, highest 5 years time who will take our place?” (Interview13).*

Participants also expressed concern over the lack of recognition of mental health professionals, and the reason many people prefer to train in other fields of health care. They observed that while other specialists working in general hospital settings receives more incentives such as call duty (extra pay), hazard allowances and even in-service trainings, they do not receive any special benefits. All the participants observed that they face challenges that are specific to working in a psychiatric environment and indicated that they needed some form of compensation. A Nurse Administrator explained: “*There are no incentives to encourage people to work here, nobody want to train in mental health care or psychiatry because there are no incentives, why should they come and endanger their lives, these are the challenges people are not interested to work here because there are no incentives” (Interview 15).*

### Physical capital

Majority of the participants expressed concern on the poor status of the facility in spite of the huge resources generated by the State from oil revenue. Physical capital such as, hospital beds, equipment, outpatient care, rehabilitation facilities, community outreach clinics, and other non-health infrastructures that are provided to promote mental health of the population are absent. A CNO elaborates *“We have gone to the ministry and met with the commissioner several times but at the end of the day nothing happens because I think these issues has not being brought to the notice of the governor. The State has the resources to provide whatever we need, yes everything…okay go and see what government is doing in other hospitals, you wouldn’t believe…so what happen to psychiatry, same old story” (Interview 1).*

All the participants indicated that the mental health facility is too small to cope with the number of patients, and overcrowding is hampering their ability to work. They observed that this has also places additional burden on staffs who are stressed, with the high patient loads and lack of resources to provide adequate levels of care. As expressed by this CNO: “*Resources to meet the needs of patients are lacking, the wards are not maintained, the out-patient clinic is small and not conducive for the large patients’ turnout, you can see patients overflow outside the premises. Basic things to provide care are not easily available, such as water, electricity, mattresses, bed sheets, toilet facilities for out-patients and so forth. We can’t improvise some of these items…we are scarifying so much for their care” (Interview 6).*

### Consumables

The majority of participants reported that the supply and pricing of psychiatric medications is regulated by the state government and the associated cost subsidized. All follow-up appointments are carried out in this facility, including doctors’ appointments and prescription repeats, with enough psychotropic medications resulting in all categories of mental illness being relatively well managed. However, as patients not only incur expenses getting to the hospital but having to pay for the medications, the costs make it difficult for most of the patients to pay and continue to stay in treatment. A Nurse Administrator illustrates: “*Clients buy their medications, the price of the drugs are not the same. Depending on the prescription, some will pay as low as N500 ($5) while others may pay as much as N10, 000 ($100) for one month. The inpatients actually spend a lot…it’s very difficult for most of them to meet up with the cost” (Interview 11).*

### Social capital

The participants observed there are no ongoing support groups throughout the state that provides support for clients and their families. However, they are a few churches and women organizations that come occasional to pray and donate clothes and food items for in-patients. A CNO elaborated: “*There are no supports for clients, in terms of government or NGOs, local or international. I have not seen any except some religious organizations who comes in to donate food and cloths during Christmas and some festive periods, many patients are left at the mercies of their families” (Interview 17).*

The majority of the participants expressed dismay over the general societal neglect of clients and families living with mental disorders. They reported that there are no welfare nets nor do any official or family associations exist to promote mental health or provide support. A Nurse Administrator observed: *“I believe if these clients get some form of support, their health can be improved. The absence of welfare net is detrimental, without resources how can they sustain the treatment? Most of them don’t have the means, it’s so sad to know the difficulties they encounter to sustain treatments” (Interview 2).*

## Discussion

The implications of these findings will be discussed, with the Context Domain first, followed by the Resource Domain.

### Context domain

Mental health care professionals suffered stigma and discrimination from within the Ministry of Health at both policy and professional level, as well as from the public. These negative societal perceptions towards mental health services and those with mental disorders, accompanied with low levels of knowledge about positive treatment outcomes have been reported in other studies [10,24,25]. Stigma attached to mental disorder has being identified as one of the hindrance to delivery of mental health service and provision of care [10,26-28]. Byrne [26] defined stigma “as a sign of disgrace or discredit, which sets a person apart from others” while Sartorius [28] defined stigma of mental disorder “as the negative attitude (based on prejudice and misinformation) that is triggered by a marker of illness e.g. odd behavior or mention of psychiatric treatment in a person’s curriculum vitae”. Stigma and discrimination associated with mental disorders are common in Nigeria, mostly associated with local belief system regarding the causes of mental disorders [29-32]. Evidence suggest stigma and discrimination is pervasive amid public and health care providers and at the level of policy makers, and that mental health professionals feel stigmatized by other staff due to the nature of their work [27,28,33].

Health professionals are reluctant to take up psychiatric practice due to stigma and a lack of institutional support, this being similar to a study of medical students’ attitude in Nigeria [24] where only 2% showed a preference for psychiatric practice. This was due to its low prestige and status of psychiatry as a profession despite the fact that more than 75% had positive views on the efficacy of psychiatry. Nurses reported that many people in need of mental health care avoid going to the psychiatric hospital for fear of being labeled. The finding supports another study in Nigeria, where 53% of the general health care workers in a teaching hospital with a psychiatric unit in the hospital did not want their office near it [30].

Despite policies being in place, there remains a lack of mental health services at PHC level at which rural communities could access information, treatment and repeat prescriptions. This is confirmed by previous studies that emphases the need to increase coverage of mental health service [34,35]. The hospital provides services for people beyond its catchment area of Rivers State, placing a strain on its ability to meet the needs of intended population, let along the additional patients. Evidence has shown that populations with high rates of socioeconomic deprivation have the highest need for mental health care and the best way to extend mental health care to the population is decentralize care [4,36], as a result developing formal community and hospital-based mental health services is crucial for their clients’ quality of life [2,7,37]. Policies should therefore implement primary mental health care in rural communities to improve access to care, uptake of treatment and, thereby reduce the burdens associated with mental disorders.

The absence of a Mental Health Coordinators and Directors in the Ministry of Health to overseer mental health activities and programs contributes to poor governance, implementation, and the low priority status of service delivery. The evidence suggests that the scarcity of public-health perspectives in mental health leadership, and the prevailing public-health priority agenda and its effect on funding are some of the barriers in mental health service [35,36,38]. To ensure good governance of the mental health care system, mental health director and coordinator positions should be established to facilitate mental health activities and programs in the Ministry of Health.

The staff felt that there was poor knowledge and understanding of mental disorders and positive treatment outcomes in the general population, this being consistent with previous study in Nigeria which shows that 96.5% of people had poor knowledge of causation of mental illness [31]. A greater awareness among the population about the causes of mental disorders and the appropriate treatment pathways could assist in creating a demand for services at all levels.

### Resources domain

Mental health services are structurally disadvantaged in Rivers State, with inadequate financing which hinders mental health delivery in the state. This finding is supported in a study in Nigeria where resources allocated to mental health service delivery were inadequate at the federal level, being allocated less than 1% of the total health budget [37]. Consistent with previous studies, out-of-pocket payment for mental health service was considered detrimental and unsatisfactory, because severe mental disorders can lead to heavy financial expenditure, create inequitable access to treatment [38,39]. Participants observed that the poor funding of services is due to policy–makers holding people with mental illnesses in low regards, these findings being supported by a study in Zambia [24], where mental illness was seen as self inflicted, resulting in discrimination at the level of government and policy. Evidence have shown that providing a quality mental health service requires proper financing and that political will is needed to ensure that accessible and humane mental health services are provided [1,2,12,36].

The human resources at the hospital are inadequate to provide the required mental health care services. Human resources shortages, an aging mental health care population and the lack of incentives for professionals was seen as challenges faced by the mental health service, this being supported by other studies [2,40,41]. Research has shown that human resources are the most valuable asset of any mental health service, and that efficient service provision relies on competent and motivated mental health personnel to provide the required services and increase the access of underserved population [12,36]. Human resource training within government is essential as is the need to make psychiatric practice attractive to other health professionals, similar financial rewards need to be offered as with other specialized areas of care.

The lack of amenities and resources is hampering its ability to provide the required services at the hospital. Since its establishment in 1977, no renovations have been carried out to address the increased use of the facility. Evidence suggest that providing efficient mental health service requires developing and maintaining infrastructure, and providing basic amenities to deliver service [5,6,12]. This lack of resource allocation over the years indicates that it should be given priority status on the public health agenda**.**

All consumable are supplied and priced in the hospital, and the facility generally holds enough psychotropic medicines for all categories of mental illness. However, the cost of purchasing these medications from the hospital is a challenge to most clients and their families. These finding supports a study of cost-effectiveness of an essential mental health intervention package in Nigeria [37] which showed cost-effective interventions using older antipsychotic drugs combined with psychosocial treatment produced one extra year of healthy life at a cost of less than US $320 per year, which is the average per capita income in Nigeria. The resource for mental health services are scarce and unevenly distributed between urban and rural communities in many developing countries, with people often relying on out-of-pocket payments [5,36,39]. Policy changes need to be carefully costed to make mental health services affordable to those seeking care.

Social capital is the resources that are available to manage mental health between individuals and formal or non formal organizations [20,28]. Collaboration among individuals, communities and formal institutions for mutual benefits is currently absent in the State. Evidence indicates that people with mental disorders benefit from drawing on community resources for support, such as consumer and family associations, formal and informal resources of family, friends, and other social networks [36,42]. Research has shown that a social support net can improve the quality of life, with better mental health outcomes and with enough collective weight, can influence policy changes [43,44]. Policies should therefore build, support and strengthen social organizations to facilitate the wellbeing of persons and families with mental illness.

The Townsend et al. [20] conceptual framework met the study objective of exploring mental health nurses’ experiences within the current mental health policy environment in Rivers State, as the tool highlighted difficulties and challenges of mental health services delivery. The use of the temple is recommended for further studies to evaluate mental health care services in other regions of Nigeria.

### Limitations

The main limitation was the small sample size and the fact that they were recruited exclusively from the neuropsychiatric Rumuigbo Hospital. However, as the intention was to assess the experiences of mental health nurses providing mental health care service within the current mental health policy environment in River State, it was not possible to include anyone else due to the lack of additional mental health services. The results may therefore not reflect other regions of the country where there are more federal funded psychiatric services. The strength of this study was the use of in-depth interviewing which provided comprehensive understanding of mental health care delivery service from the perspectives of mental health nurses.

### Recommendations

The following recommendations are made to improve health care services for persons with mental illness and their families in River State in Nigeria:

● Stigma and discrimination of mental illness by government officials and staff needs to be addressed at all levels.

● Education about the causes and treatment pathways for mental illnesses needs to happen within government departments and to the population at large.

● Public health services should include community-based care to reduce costs of accessing services and allow affected people to continue to stay in treatment.

● Specialized psychiatric training of health professionals need to be provided by the Ministry of Health to overcome the current human resources shortage, and the aging health population, and to make provision for future growth in this sector.

● Incentives need to be provided to attract health professionals into the discipline.

● Psychotropic medications should be decentralized from psychiatric hospitals to other health care facilities where there are trained nurses and general practitioners to enable clients to refill prescriptions locally.

● Social welfare nets for persons with mental disorders should be provided and support by trained professionals at all levels of care.

## Conclusion

The current model of institutionalized mental health care in River State not only results in insufficient care to the communities it serves, it also impacts negatively on the experiences of the health professionals who provide the care. The lack of resources for community based services and information at all levels prevents people from seeking the advice and treatment they need, and results stigma and discrimination of affected persons and families. In executing its mandate, the Ministry of Health needs to implement the policies it has developed, appoint appropriate directors, train the necessary staff and provide the resources to enable these services to be put in place. Failure to address the needs of the staff will result in their continued low morale, depleting mental health human resources and the marginalization and stigmatization of people affected by the disease.

## Competing interest

We declare that there is no competing interests.

## Authors’ contributions

(1) IOJ: Conceptualization of the study, literature search, data collection, analysis of data and drafting of the article and finalization. (2) LRU: Conceptualization of study, analysis of data, critical revision of the article and approving the final version. (3) LEM: Conceptualization of study, analysis of data, critically revising article, approving final version.
